# Autism in Toddlers: Can Observation in Preschool Yield the Same Information as Autism Assessment in a Specialised Clinic?

**DOI:** 10.1155/2013/384745

**Published:** 2013-02-07

**Authors:** Gunilla Westman Andersson, Carmela Miniscalco, Ulrika Johansson, Christopher Gillberg

**Affiliations:** ^1^Gillberg Neuropsychiatry Centre, Sahlgrenska Academy, University of Gothenburg, Kungsgatan 12, 411 19 Gothenburg, Sweden; ^2^The Queen Silvia Children's Hospital, Otterhällegatan 12 A, 411 18 Gothenburg, Sweden

## Abstract

We wanted to know whether preschool observation of children suspected of suffering from autism can provide the same information about core autism symptoms as the Autism Diagnostic Observation Schedule (ADOS) performed in a clinic. Forty 2–4-year-old children (9 girls, 31 boys), referred for assessment of suspected autism spectrum disorder participated in the study. The symptom areas covered by the ADOS algorithm were scored by an education specialist after free-field observation of each child in the preschool without using the prescribed ADOS materials. The ADOS was then completed in a clinic setting by examiners blind to the preschool results. Excellent agreement across results obtained at the two different types/settings of observations was found. The only significant difference found was with regard to spontaneous initiation of joint attention. The present study does not address the issue of whether or not one of the methods used is superior to the other when it comes to determining the “true” level of “autism problems” in these children. However, it is of interest that free-field preschool observation of children with suspected autism using a structured checklist yields very similar information as that obtained at ADOS assessment performed in a clinic setting.

## 1. Introduction

Autism spectrum disorder (ASD) has symptom onset early in life and a prevalence of about one percent of the general population [1]. ASD involves severe and pervasive restrictions regarding reciprocal social interaction, social communication, and imagination/behaviour and occurs at all levels of cognitive functioning. Most children with ASD have problems with generalisation, which affects their behaviour in different contexts. Young children with ASD have more nonfunctional and repetitive play than typically developing children [2], and impairment in play, imitation, and joint attention are important predictors of autism [3, 4]. Systematic research has highlighted the importance of early intervention for children with ASD [5–7]. It follows that early detection is crucial and that valid assessment tools designed for young children (and taking possible gender differences into account) are needed [8, 9].

One of the most widely advertised and used autism assessment tools is the Autism Diagnostic Observation Schedule (ADOS) [10]. The ADOS is a standardised, semistructured instrument, shown to be valid for a clinical diagnosis of autism [11]. It is intended for use in a structured clinical setting. There are four different modules, depending on the level of expressive language (ranging from preverbal to fluent speech). For young children, module 1 is used for nonverbal children and module 2 for children with phrase speech. Module 3 is used for older children with fluent speech, and module 4 is intended for verbally fluent adolescents and adults. The ADOS is intended for use by specially trained professionals in the clinic. Observations of communication, social interaction, play and imagination, and stereotyped behaviours/interests are made in a play/interaction situation using structured activities and materials/toys. One test manager interacts with the child, and usually one professional observes the child during the test, which takes about 30–50 minutes. Immediately after the ADOS procedure both professionals, that is, the test manager and the observer, score the child's performance together according to the manual. An algorithm covering 17 different autism-related areas for module 1 and 16 areas for module 2 is used, and the scoring result provides a cutoff for diagnosis at various levels of ASD, based on the total score for communication and reciprocal social interaction problems. 

A few other observational instruments have recently been reported to have potential for the diagnostic assessment of autism in young children. One of these, the Classroom Observation Schedule to Measure Intentional Communication (COSMIC) [12], focuses on communication in natural settings. Relevant items from the COSMIC showed significant associations with the five selected corresponding items on the ADOS, and Interrater reliability was high. The items from the ADOS were (1) overall level of nonechoed language, (2) echolalia, (3) pointing, (4) gestures, and (5) spontaneous initiation of joint attention. Another recently reported instrument, the Playground Observation Checklist (POC) [13], discriminated in respect of social behaviour between children with autism, mental retardation, and typical development. However, no comparison with the ADOS was made. Both the COSMIC and the POC were used with children aged 4–11 years who had been clinically diagnosed with autism before the studies were performed. 

According to a newly published report from the Swedish Council of Health Technology Assessment (SBU) there is a great need for further knowledge and development of diagnostic instruments regarding ASD and other neuropsychiatric disorders [14]. There is a particular need to further develop and evaluate methods for ASD observation in the child's everyday environment such as in day nurseries, preschools, and classrooms. We need instruments that can be used in order to identify symptoms of autism even if the child, for whatever reason, cannot participate in a formal test situation at the clinic and to establish whether or not it would be possible to “pick up” or make a preliminary diagnosis of autism even in the absence of full assessment in a clinical setting. This would also be important for epidemiological studies, where “quick and dirty,” but ecologically valid, instruments are much needed. Clinical experience suggests that naturalistic observation of the child with suspected ASD in the “natural” environment of his/her preschool and observation in the clinic using the ADOS, often provides additional information about the child. This is also emphasised in the diagnostic manuals, including the DSM-IV [15]. 

The aim of this study is to determine whether structured observation (of free-field behaviour) in a preschool setting of 2–4-year-old children suspected of suffering from ASD, yields the same overlapping or different information as the ADOS used in a specialised autism clinic?

## 2. Method

The study was conducted within the AUDIE project (AUtism Detection and Intervention in Early life) [16]. The aim of the AUDIE is to detect toddlers in the general population with suspected ASD/other developmental disorders, make comprehensive clinical assessments, and provide early intervention. In brief, all 30-month-old Gothenburg children are screened for language, communication, and ASD problems in well-baby clinics. All children screening positive are referred for ASD in-depth assessment to the Child Neuropsychiatry Clinic (CNC), which is a local, regional, and nationwide clinic for assessment of ASD and other Early Symptomatic Syndromes Eliciting Neurodevelopmental Clinical Examinations (ESSENCE) [1]. 

### 2.1. Participants

Forty children (9 girls, 31 boys), aged 29–51 months (mean age 40 months) (Table 1), participated in the study. They had all been referred to the CNC for suspected ASD. These 40 children were consecutively referred through the AUDIE project with a clinical referral diagnosis of suspected ASD and who regularly attended a preschool (*n* = 39) or another day-care facility group that included several other children (*n* = 1). 

### 2.2. Diagnostic Assessment at the CNC

As part of the AUDIE project, all children underwent the following assessments: (a) medical-neurological-psychiatric examination of the child; (b) child and family medical/psychiatric history taken from parent; (c) Griffiths' Developmental Scales [17] and whenever appropriate according to developmental age of the child the Wechsler Preschool and Primary Scale of Intelligence-Revised (WPPSI-R) [18]; (d) Vineland Adaptive Behavior Scales (VABS) [19]; (e) MacArthur Communicative Development Inventory [20, 21] and the Reynell Developmental Language Scales III [22]; (f) Diagnostic Interview for Social and Communication Disorders (DISCO-11) [23]; (g) ADOS; and (h) preschool observation in accordance with a newly constructed protocol developed for the present study (see below). The professionals included in the CNC team were (a) a physician; (b) a neuropsychologist; (c) a speech and language pathologist; and (d) a special education teacher. 

All the various assessments (a) through (h) were performed independently of each other, and the research clinicians remained blind to other assessors' results until the conjoint diagnostic case conference, which was held after the completion of all assessment as listed under from (a) to (h). At this conference, the assessment team made consensus clinical diagnoses according to the DSM-IV criteria for disorders first evident in childhood or adolescence, on the basis of all available information. As regards ASD/PDD, the participating children in the present study were clinically diagnosed as autistic disorder (AS) (*n* = 22), other ASD (*n* = 12), and non spectrum (NS) (*n* = 6) (Table 1). Note that these diagnoses were only made *after* the preschool observation and ADOS assessments had been completed.

### 2.3. ADOS Assessment at the CNC

Two special education teachers (examiner 1 (GWA) and examiner 2 (UJ)) performed the ADOS-G assessments at the clinic. To avoid bias, examiner 1 performed the preschool observation of child 1 who was then (blindly) assessed by examiner 2 using the ADOS in the clinic together with another observer. Examiner 2 then performed the preschool observation of child 2 who was (blindly) ADOS assessed by examiner 1 in the clinic together with another observer. All ADOS clinical assessments were videotaped in order to perform reliability measures and were scored by the examiner and the observer together.

### 2.4. Preschool Observation according to a New Structured Protocol

In order to allow reasonable comparisons to be made, the “symptom areas” covered by the ADOS algorithm items were used as a “template” for the construction of the preschool observation checklist that would be used by a special education teacher with long-term experience of autism, and trained in the use of ADOS (see Appendix at the Supplementary Material available online at http://dx.doi.org/10.1155/2013/384745). These areas included in the ADOS algorithm were used both in the clinic and in the preschool. The examiner was aware that the observed child was under assessment for suspected ASD, but other than age and gender, the examiner was “blind” and had no further information about the child at the time of observation.

The preschool teachers were instructed to be around the children as they normally would in everyday indoor situations. The “ADOS-similar” observations were made mainly in group activities and free play. If the child did not spontaneously perform activities, allowing observation of a particular area, the examiner herself interacted with the child, presented the task to her/him, or asked the teacher to do so. The classrooms were designed for typically developing children, and the number of children in the groups ranged from 15 to 30 children. No ADOS-specific materials were used; instead all material used in this observation belonged to the preschool. In other words, only the symptom areas checked during the preschool observation were the same as those scored using the ADOS. The observation took about an hour to perform and was scored in accordance with the ADOS algorithm. All completed preschool observation research protocols were sealed and stored away, so that other research clinicians could not take part of the results until the final conjoint diagnostic assessment was made. 

### 2.5. Interrater Reliability

Interrater reliability was calculated as percent agreement using the point-by-point method and as weighted kappa [24], calculated in MedCalc version 10.2 [25].

Interrater reliability between the two examiners in the preschool observations was calculated on all the variables in the ADOS algorithm for communication, reciprocal social interaction, play and behaviour/interests, of the preschool observation results for 10 children. These children were included in the larger AUDIE project, but not in the present study. Examiner 1 and 2 observed the same child at the same time at preschool and scored according to the protocol (see Appendix at the Supplementary Material available online at http://dx.doi.org/10.1155/2013/384745), not talking to each other about what they observed. To measure the Interrater reliability of the clinical ADOS examination, another 10 children were blindly examined using videotapes of the ADOS assessment. Examiner 1 (blindly) examined 5 videotaped observations, performed “live” by examiner 2, and examiner 2 (blindly) examined 5 videotaped observations performed by examiner 1 (Table 2).

### 2.6. Statistics

The Wilcoxon signed rank test was used to compare child behaviours in preschool and clinic. There were some methodological challenges stemming from the fact that 24 children were coded using module 1 (preverbal), and 16 were coded using module 2 (phrase speech). We analysed the data in different ways to ensure that the conclusions do not depend on how we handled differences across instruments. Specifically, ADOS modules 1 and 2 contain 11 common variables. In addition, in module 1 there are another 6 variables unrelated to the common ones, and in module 2, there are 5 such unrelated variables. This is shown in Table 3. The material was analysed in three different stages.

1. Comparison of the overall results of each domains of modules 1 and 2 and the combined result of communication and reciprocal social interaction, which, in ADOS, gives cutoff for diagnosis. Thus, no attempt was made to correct for differences in the modules.
2. To get a larger number of comparable variables, the overall summarised results of only the common variables for both module 1 and 2 were calculated. This score will be referred to as the “collapsed global” score. Children were compared also on this score from the preschool observation and from the ADOS assessment.
3. Each variable within each domain was analysed.

Note that numbers of variables in Table 3 vary depending on whether the item belonged to module 1 (*n* = 24), module 2 (*n* = 16) or was shared by module 1 and module 2 (*n* = 40).

### 2.7. Ethics

The study was approved by the Human Ethics Committee at the Medical Faculty at the University of Gothenburg, Sweden. Informed consent was obtained from at least one of the parents/responsible carers in each case. 

## 3. Results

### 3.1. Reliability Results

The results are shown in Table 2. For Interrater reliability for preschool observation, the percent agreement ranged from 83% to 94%, and weighted kappa statistics ranged from 0.82 to 0.93. For Interrater reliability on the ADOS, percent agreement ranged from 88% to 100%, and weighted kappa ranged from 0.85 to 1.0. Interrater reliability measures were considered good to very good.

### 3.2. Study Results

In Table 3 data from both module 1 and module 2 in ADOS are presented for all children divided into four domains: (1) communication, (2) reciprocal social interaction, (3) play and imagination, and (4) stereotyped behaviours and restricted interests. The ADOS clinical and the preschool observation both showed a mean result of more than 12 points in combined total score for communication and reciprocal social interaction (Table 4), indicating a diagnosis of autism according to ADOS algorithm, at least at group level. Sign test comparisons of the variables rated in preschool and corresponding items in the clinic showed a significant difference only with regard to spontaneous initiation of joint attention (*P* = 0.0129). For all other observed variables there was good agreement according to sign test, percentage agreement, and weighted kappa across the two methods and the two settings. In some cases the score was somewhat higher in ADOS clinical, and in some cases it was higher in the preschool observation. This is shown in the “ADOS higher” and “preschool higher” columns in Table 3.

## 4. Discussion

The main finding of this study was that preschool observation by an autism-experienced rater of children with suspected ASD, yielded almost the same amount and type of information, as highly structured ADOS assessment performed by two specially trained clinicians in a specialised clinic setting. Initiation of joint attention, suggested to be one of the key difficulties in young children with ASD [3, 4], was the only domain where the ADOS at the clinic indicated more problems than preschool observation of the child in interaction with typically developing children. However, based on the results of the present study we cannot determine which of the two observation settings is more informative about the child's “true” level of joint attention. 

Unlike in the study of COSMIC [12] and the POC [13], the researchers remained blind to the children's diagnosis when the observations were made, and our participants were of considerably younger age. Another contrast to the COSMIC study is that we used the same symptom areas, but in different contexts. 

The findings, if confirmed by other researchers, suggest that preschool observation using the protocol included here (which is not equivalent to that of the ADOS, albeit covering the same areas) and performed by ASD experienced examiners could be used for rating observable autism symptoms. This could have important implications for field trials and epidemiological studies of autism, but also for autism diagnostic services, for example, in rural and sparsely populated areas. While preschool observation entails cost for travel for the examiner (including time costs), ADOS observation at the clinic often consists of two specially trained experts resulting in financial costs for both clinic and family, as well as inconvenience for the parents involved. However, in other instances the clinic ADOS assessment could be a more efficient and effective assessment tool than preschool observation. Conclusions and recommendations in this respect would have to be made on an individual basis.

Further, at the preschool visit one gets information about the child, that is not included in the clinic ADOS, for example, how the child can handle different situations in daily life. Some of this information may actually be even more important than the diagnosis of autism per se [15]. However, it is important to note that scoring above an algorithm cutoff is not the same as actually “getting” a diagnosis. It is crucial to interpret results from ADOS and preschool observations in relation to other information obtained at comprehensive neuropsychiatric assessment, including child and family medical/psychiatric history taken from parent, developmental quotient, and language measures.

Although we found very strong agreement across the two assessment methods, and even though we realise that this could be taken as support for an either/or approach in the delivery of diagnostic long-term clinical services, our experience suggests that in clinical practice, flexibility is important. Given that every child with ASD is a unique individual, one needs to remain open for individualisation, even in clinics where there is an agreed core protocol for ASD assessment. Preschool teachers, often have a high level of knowledge about the child, and this is important to take advantage of in the ASD diagnostic process. Preschool teachers should be encouraged to make observations and documentations of the child in everyday situations, so as to better enable identification of the child's strengths and difficulties. It is crucial that teachers in preschool receive information and formal training about children with ASD. When preschool teachers have good ASD “know-how,” their commitment will be much greater in terms of early intervention in the preschool setting [6, 7, 26].

### 4.1. Limitations

There was no comparison group, so we do not know how typically developing children would be scored at this type of ASD assessment. However, the aim of this work was to *compare two settings* for an observation aiming to detect ASD symptoms and signs, and it was *not* intended to be *a comparison of participants' problems*. A larger study group would have been preferred, but the constraints of the AUDIE project did not allow inclusion of more cases. 

### 4.2. Further Research

This study is focused on preschool children only. This means that we know nothing about what the result would be for older children. It would be valuable to perform similar studies in children with suspected ASD at older ages. It would also be important to perform a confirmatory study including a larger number of participants, not least so as to enable comparison of girls and boys. The ADOS severity metric [27] is a tool that could be useful for these comparisons. Finally, it would be of interest to determine the relative predictive validity of preschool observation as against ADOS performed in the clinic in respect of the “final” ASD consensus diagnosis.

## Supplementary Material

Appendix 1: Pre-school observation Module 1.
Appendix 2: Pre-school observation Module 2.

## Figures and Tables

**Table 1:** Participants by module, age, gender, and clinical diagnosis. Module 1 = preverbal, module 2 = phrase speech.

Module	Mean age (months)	Girls	Boys	AS	ASD	NS	Total number of individuals
1	38	5	19	20	3	1	24
2	42	4	12	2	9	5	16

Total	40	9	31	22	12	6	40

**Table 2:** Results of Interrater reliability measurements of ADOS (*n* = 10) and preschool observation (*n* = 10). Calculated in percent agreement—point-by-point method and weighted kappa.

	Interrater measurement	
	Percent agreement	Weighted kappa
ADOS		
Communication	88	0.85
Reciprocal social interaction	93	0.91
Play and imagination	100	1.0
Stereotyped behaviours and restricted interests	90	0.89
Preschool observation		
Communication	90	0.89
Reciprocal social interaction	94	0.93
Play and imagination	88	0.82
Stereotyped behaviors and restricted interests	83	0.82

**Table 3:** Agreement between ADOS and preschool observation findings (module 1, *n* = 24; module 2, *n* = 16). Number of higher score in each type of observation is described.

Domains	*N*	Agreement	ADOS higher	Preschool higher	*P* value	Weighted Kappa
Communication						
Frequency of vocalization directed to others	24	17 (71%)	4 (17%)	3 (13%)	1.0000	0.33
Amount of social overtures	16	10 (63%)	5 (31%)	1 (6.3%)	0.2188	0.35
Stereotyped/idiosyncratic use of words or phrases	40	28 (70%)	4 (10%)	8 (20%)	0.3877	0.43
Use of others body to communicate	24	13 (54%)	7 (29%)	4 (17%)	0.5488	0.26
Conversation	16	9 (56%)	2 (13%)	5 (31%)	0.4531	0.38
Pointing	40	24 (60%)	10 (25%)	6 (15%)	0.4545	0.52
Gestures	40	23 (58%)	9 (23%)	8 (20%)	1.0000	0.44
Reciprocal social interaction						
Unusual eye contact	40	32 (80%)	4 (10%)	4 (10%)	1.0000	0.56
Facial expressions directed to others	40	25 (63%)	6 (15%)	9 (23%)	0.6072	0.53
Shared enjoyment in interaction	24	12 (50%)	3 (13%)	9 (38%)	0.1460	0.21
Showing	24	15 (63%)	6 (25%)	3 (13%)	0.5078	0.41
Spontaneous initiation of joint attention	40	26 (65%)	12 (30%)	2 (5.0%)	0.0129	0.57
Response to joint attention	24	13 (54%)	4 (17%)	7 (29%)	0.5488	0.42
Quality of social overtures	40	25 (63%)	4 (10%)	11 (28%)	0.1185	0.47
Quality of social response	16	7 (44%)	2 (13%)	7 (44%)	0.1797	−0.07
Amount of reciprocal social communication	16	10 (63%)	3 (19%)	3 (19%)	1.0000	0.33
Overall quality of rapport	16	8 (50%)	3 (19%)	5 (31%)	0.7266	0.35
Play and imagination						
Functional play with objects	24	15 (63%)	4 (17%)	5 (21%)	1.0000	0.43
Imagination/creativity	40	26 (65%)	8 (20%)	6 (15%)	0.7905	0.57
Stereotyped behaviours and restricted interests						
Unusual sensory interest in play material/person	40	30 (75%)	6 (15%)	4 (10%)	0.7539	0.14
Hand and finger and other complex mannerism	40	26 (65%)	6 (15%)	8 (20%)	0.7905	0.51
Unusual repetitive interests or stereotyped behaviours	40	22 (55%)	8 (20%)	10 (25%)	0.8145	0.42

The comparison data is presented as *n* (%).

The *P* values are calculated using a Sign test.

**Table 4:** Comparison between the total score in the different domains of preschool observation and ADOS.

Domains	Preschool M (SD) Min–max	ADOS M (SD) Min–max	Differences M (SD) Min–max	*P* value
Total: communication (*N* = 40)	4.13 (2.46)	4.50 (2.42)	−0.38 (1.86)	0.1034
	0.00–10.00	0.00–9.00	−4.00–6.00	
Module 1 (*n* = 24)	5.33 (2.22)	5.71 (2.10)	−0.38 (1.95)	0.1564
	1.00–10.00	2.00–9.00	−3.00–6.00	
Module 2 (*n* = 16)	2.31 (1.54)	2.69 (1.62)	−0.38 (1.78)	0.4785
	0.00–6.00	0.00–7.00	−4.00–3.00	
Total: reciprocal social interaction (*N* = 40)	8.13 (4.33)	7.70 (4.26)	0.43 (2.70)	0.4196
	0.00–14.00	0.00–14.00	−5.00–6.00	
Module 1 (*n* = 24)	10.21 (3.67)	9.71 (3.86)	0.50 (2.72)	0.5178
	1.00–14.00	2.00–14.00	−5.00–6.00	
Module 2 (*n* = 16)	5.00 (3.29)	4.69 (2.85)	0.31 (2.75)	0.6573
	0.00–10.00	0.00–10.00	−4.00–5.00	
*Combined total; communication and reciprocal social interaction* (*N* = 40)	12.25 (6.56)	12.20 (6.37)	0.05 (3.85)	0.7180
	0.00–23.00	1.00–23.00	−7.00–12.00	
Module 1 (*n* = 24)	15.54 (5.52)	15.42 (5.50)	0.13 (3.76)	0.5574
	2.00–23.00	6.00–23.00	−7.00–12.00	
Module 2 (*n* = 16)	7.31 (4.67)	7.38 (4.19)	−0.06 (4.11)	1.0000
	0.00–16.00	1.00–17.00	−7.00–7.00	
Total: play and imagination (*N* = 40)	2.08 (1.65)	2.08 (1.62)	0.00 (1.04)	1.0000
	0.00–4.00	0.00–4.00	−3.00–4.00	
Module 1 (*n* = 24)	3.13 (1.23)	3.04 (1.30)	0.08 (1.21)	0.8418
	0.00–4.00	0.00–4.00	−3.00–4.00	
Module 2 (*n* = 16)	0.50 (0.63)	0.63 (0.72)	−0.13 (0.72)	0.7266
	0.00–2.00	0.00–2.00	−1.00–1.00	
Total: stereotyped behaviours and restricted interests (*N* = 40)	1.83 (1.39)	1.83 (1.41)	0.00 (1.26)	0.9660
	0.00–5.00	0.00–5.00	−3.00–2.00	
Module 1 (*n* = 24)	2.21 (1.50)	2.25 (1.51)	−0.04 (1.37)	0.9089
	0.00–5.00	0.00–5.00	−3.00–2.00	
Module 2 (*n* = 16)	1.25 (1.00)	1.19 (0.98)	0.06 (1.12)	1.0000
	0.00–3.00	0.00–3.00	−2.00–2.00	
*Collapsed global score (module 1 and module 2) *				
Communication	2.23 (1.67)	2.28 (1.45)	–0.05 (1.18)	0.8179
	0.00–6.00	0.00–5.00	−3.00–3.00	
Reciprocal social interaction	4.58 (2.89)	4.60 (2.62)	–0.03 (1.54)	0.8876
	0.00–8.00	0.00–8.00	–5.00–4.00	
Total; communication and reciprocal social interaction	6.80 (4.33)	6.88 (3.91)	–0.08 (2.34)	0.3980
	0.00–13.00	1.00–13.00	–5.00–7.00	
Play and imagination	1.25 (0.84)	1.28 (0.85)	–0.03 (0.66)	1.0000
	0.00–2.00	0.00–2.00	–1.00–2.00	
Stereotyped behaviours and restricted interests	1.83 (1.39)	1.83 (1.41)	0.00 (1.26)	0.9660
	0.00–5.00	0.00–5.00	–3.00–2.00	

The data is presented as mean (SD)/Min–Max.

Differences are preschool values minus ADOS values.

The *P* values are calculated using a Wilcoxon signed rank test.

Collapsed global scores only include tests involving both modules.
