# Supplementary material for: Autism in Toddlers: Can Observation in Preschool Yield the Same Information as Autism Assessment in a Specialised Clinic?
Source: ScientificWorldJournal. 2013 Feb 7;2013:384745. doi: 10.1155/2013/384745 (PMC3582094; doi:10.1155/2013/384745)
Supplement: Supplementary file 1 — Appendix 1: Pre-school observation Module 1. Appendix 2: Pre-school observation Module 2. [file 384745.f1.doc]

# Pre-school observation Module 1

From the algorithm in ADOS

**Name:________________________________
Year of birth:_____________
Date:________________
Examiner:____________________________

Communication Points**Frequency of vocalization directed to others _________________________________
Stereotyped/idiosyncratic use of words or phrases _____________________________
Use of others body to communicate ________________________________________
Pointing ______________________________________________________________
Gestures______________________________________________________________
 **Communication total ______________


Reciprocal social interaction Points**Unusual eye contact _____________________________________________________
Facial expressions directed to others ________________________________________
Shared enjoyment in interaction ___________________________________________
Showing ______________________________________________________________
Spontaneous initiation of joint attention _____________________________________
Response to joint attention ________________________________________________
Quality of social overtures ________________________________________________
 **Social interaction total ________

 Communication + social interaction total_________


Play and imagination Points**Functional play with objects _______________________________________________
Imagination/creativity ____________________________________________________
 **Play/imagination total** ____

**Stereotyped behaviours and restricted interests Points**Unusual sensory interest in play material/person _______________________________

Hand and finger and other complex mannerism________________________________
Unusual repetitive interests or stereotyped behaviours___________________________
 **Stereotyped behaviours and restricted interests total_________**

**The items should be scored using the same metric and criteria as the ADOS.**
