# Supplementary material for: Autism in Toddlers: Can Observation in Preschool Yield the Same Information as Autism Assessment in a Specialised Clinic?
Source: ScientificWorldJournal. 2013 Feb 7;2013:384745. doi: 10.1155/2013/384745 (PMC3582094; doi:10.1155/2013/384745)
Supplement: Supplementary file 2 [file 384745.f2.doc]

# Pre-school observation Module 2

From the algorithm in ADOS

**Name:________________________________
Year of birth:_____________
Date:________________
Examiner:____________________________**
 **Communication**  **Points**Amount of social overtures/maintenance of attention ___________________________
Stereotyped/idiosyncratic use of words or phrases _____________________________
Conversation __________________________________________________________
Pointing ______________________________________________________________
Gestures______________________________________________________________
 **Communication total** _______________


**Reciprocal social interaction Points**Unusual eye contact _____________________________________________________
Facial expressions directed to others ________________________________________
Spontaneous initiation of joint attention _____________________________________
Quality of social overtures________________________________________________
Quality of social response ________________________________________________
Amount of reciprocal social communication __________________________________
Overall quality of rapport_________________________________________________
 **Social interaction total ________

 Communication + social interaction total_________


Play and imagination Points** Imagination/creativity ___________________________________________________
 **Play/imagination total** ____

**Stereotyped behaviours and restricted interests Points**Unusual sensory interest in play material/person _______________________________

Hand and finger and other complex mannerism________________________________
Unusual repetitive interests or stereotyped behaviours___________________________
 **Stereotyped behaviours and restricted interests total_______


The items should be scored using the same metric and criteria as the ADOS.**
